# Microgels Formed by Spontaneous Click Chemistries Utilizing Microfluidic Flow Focusing for Cargo Release in Response to Endogenous or Exogenous Stimuli

**DOI:** 10.3390/pharmaceutics14051062

**Published:** 2022-05-15

**Authors:** Paige J. LeValley, Amanda L. Parsons, Bryan P. Sutherland, Kristi L. Kiick, John S. Oakey, April M. Kloxin

**Affiliations:** 1 Chemical and Biomolecular Engineering, University of Delaware, Newark, DE 19716, USA; pleval@udel.edu (P.J.L.); bsuth@udel.edu (B.P.S.); 2 Chemical Engineering, University of Wyoming, Laramie, WY 82071, USA; aparso11@uwyo.edu; 3 Materials Science and Engineering, University of Delaware, Newark, DE 19716, USA; kiick@udel.edu; 4 Biomedical Engineering, University of Delaware, Newark, DE 19716, USA

**Keywords:** hydrogels, microgels, protein delivery, controlled release, microfluidics, degradable materials, stimuli responsive, click chemistry, light, reducing environment

## Abstract

Protein therapeutics have become increasingly popular for the treatment of a variety of diseases owing to their specificity to targets of interest. However, challenges associated with them have limited their use for a range of ailments, including the limited options available for local controlled delivery. To address this challenge, degradable hydrogel microparticles, or microgels, loaded with model biocargoes were created with tunable release profiles or triggered burst release using chemistries responsive to endogenous or exogeneous stimuli, respectively. Specifically, microfluidic flow-focusing was utilized to form homogenous microgels with different spontaneous click chemistries that afforded degradation either in response to redox environments for sustained cargo release or light for on-demand cargo release. The resulting microgels were an appropriate size to remain localized within tissues upon injection and were easily passed through a needle relevant for injection, providing means for localized delivery. Release of a model biopolymer was observed over the course of several weeks for redox-responsive formulations or triggered for immediate release from the light-responsive formulation. Overall, we demonstrate the ability of microgels to be formulated with different materials chemistries to achieve various therapeutic release modalities, providing new tools for creation of more complex protein release profiles to improve therapeutic regimens.

## 1. Introduction

Biologics play an important role in the treatment of numerous diseases and conditions ranging from Crohn’s disease to cancer [1,2,3]. These therapies, which include different types of bioactive proteins and cells, represent the most advanced treatments available and as such have continued to grow in market importance over the past decade [1]. While biologics have continued to grow in popularity within the medical field, there are still challenges associated with these types of therapies, including stringent storage conditions, costly therapeutic regimens, and limited options for controlled delivery when and where needed [2,3]. Toward decreasing the cost and extending therapeutic time scales, a range of therapeutic delivery strategies are being investigated. By controlling the local rate of delivery, altered pharmacokinetics and localized concentrations for the biologics cargo can be achieved over extended time periods, potentially affording improved efficacy, reduced costs, and lowered adverse side effects [4]. Liposomes [5], nanoparticles [6], microparticles [7], protein–polymer conjugates [8], and hydrogels [9] have all been designed to control and extend the release of biologics. Among these, hydrogel-based delivery platforms afford high loading-capacity of hydrophilic bioactive proteins with tunable release profiles managed through network structure and design.

Injectable hydrogels, made with synthetic or natural polymers or combinations of them, have been engineered for the controlled delivery of bioactive proteins ranging in size from growth factors (~20 kDa) to antibodies (~150 kDa) [10]. The crosslinks or polymer backbones of these networks are typically designed to degrade in response to either internal stimuli found within or external stimuli applied to biological systems. Degradation mechanisms that utilize endogenous stimuli include hydrolysis, reduction sensitivity, and enzymatic sensitivity [11]. Hydrolysis provides universal degradation of the hydrogel within aqueous, biological systems, whereas reduction and enzymatic sensitivity yield biased degradation within environments with higher reduction potential or enzyme concentrations (i.e., cancerous tissues) [4,11,12]. To achieve injectable formulations, spontaneous click chemistries [9], dynamic covalent [12], non-covalent chemistries [13], and microgels [14] have been utilized, where microgels are of growing interest for a range of applications from therapeutic delivery to bioinks and scaffolds for tissue regeneration and repair [10,15].

Microgels, defined as hydrogel particles with a diameter between 0.1 and 100 µm by IUPAC, are of particular interest for protein delivery vehicles owing to their modularity and ability to be injected as preformed hydrogel formulations [10,16]. Indeed, microgels allow decoupling of the conditions used for hydrogel formation from those used for injection, allowing a wide range of initial and temporal properties to be achieved. These properties provide improved control over network structure and thereby afford a high level of control over the stimuli-responsive behavior, leading to faster and sharper release profiles in comparison to injectable bulk hydrogels due to their high surface area to volume ratio [10,17]. Microgels have been utilized for the controlled delivery of growth factors [18], interleukins [13], and cells [19]. Their modularity affords the potential to deliver microgels formulated with different encapsulated or bound proteins, degradation rates, or mechanical or biochemical properties for achieving more complex release profiles and controllable combination therapies. However, the range of responsive chemistries and network architectures that have been integrated into microgels is limited relative to their bulk hydrogel counterparts. This is particularly the case for microgels formed with microfluidic methods, where less polydisperse microgels are formed compared to those formed using batch emulsions, suspensions, or extrusion fragmentation methods [15]. Notably, the monodisperse or homogeneous size of microgels achieved with microfluidic methods can afford consistent degradation and release profiles yet the chemistries examined to date within these systems is limited, as compared to methods for forming microgels that result in more heterogenous size distributions [20,21,22,23,24,25,26,27,28,29,30,31]. Precipitation methods for forming microgels also result in homogenously sized populations, but control over degradation chemistries has been limited due to requirements for microgel formation [32]. Broadened microgel chemical versatility is needed to more fully realize a diverse range of controlled release profiles for protein-based cargoes [33].

**Figure 1:**
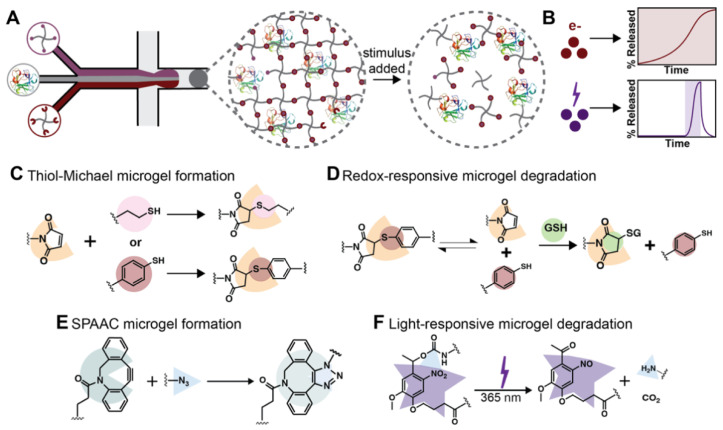
*Overview of approach.* (**A**) Microgels were formed between end-functionalized four-arm PEG macromers and containing a model biopolymer using MFF. The resulting hydrogel network encapsulated the model cargo that is released upon the addition of a stimulus. (**B**) Redox (e-labeled population) and light (lightning bolt labeled population) responsive microgel populations were formed and can be utilized to create different release profiles based on the degradable content within redox-responsive populations, for achieving tunable and sustained release, and when light is applied to the system, for achieving triggered burst release, respectively. (**C**) Thiol-Michael additions between PEG-4-maleimides and PEG-4-alkyl-thiols or PEG-4-aryl-thiols were utilized to form redox-responsive microgels. (**D**) The use of PEG-4-aryl-thiols introduces a mechanism for redox-responsive behavior through the retro-Michael addition and subsequent reaction with endogenous thiols, such as glutathione (GSH), for network degradation. (**E**) Microgels were also formed using a SPAAC reaction between PEG-4-DBCO and PEG-4-NB-azide, and (**F**) light degradability was imparted through the incorporation of NB moieties within the microgel crosslinks.

In this work, we examined the ability to form microgels responsive to endogenous and exogenous stimuli and laden with a biopolymer cargo using different spontaneous click chemistries and labile linkers within a microfluidic flow-focusing (MFF) device (Figure 1). First, microgels were formed through a thiol-Michael addition and, by altering the nature of the thiol moiety, could be made to either degrade in aqueous environments or degrade in response to glutathione (GSH, a peptide with increased concentrations in tumors) through the retro-Michael addition in combination with aqueous environments. The degradation behavior of the formed redox-responsive microgels in the presence of different buffer conditions was evaluated toward creating sustained release of large hydrophilic therapeutic proteins. Next, to establish the utility of the microfluidic device design to form microgels using different polymerization and degradation chemistries, light-responsive microgels were formed using a strain-promoted azide-alkyne cycloaddition (SPAAC). Light responsiveness was achieved through the incorporation of a nitrobenzyl (NB) cleavable moiety into the hydrogel backbone. Finally, the light-based degradation of NB-containing microgels was examined toward creating on-demand rapid release of biopolymer cargo. Together, these new microgel chemistry formulations can be utilized to control the release of hydrophilic therapeutics over multiple time scales and allow various levels of user control (days to weeks in response to endogenous stimuli or immediately with externally applied stimulus), providing a path toward improved therapeutic treatment regimens.

## 2. Materials and Methods

*Materials and General Characterization.* General organic reagents and solvents were purchased from commercial sources and used as received unless otherwise stated. Deionized (DI) water (18 million ohms-centimeter) was purified onsite using a Milli-DI water purification system (Millipore Sigma, Burlington, MA, USA). Amine end-functionalized four-arm PEG (5 kDa, PEG-4-amide) and hydroxyl end-functionalized four-arm PEG (5 kDa, PEG-4-OH) were purchased from JenKem Technology USA Inc. (Allen, TX, USA). Maleimide end-functionalized four-arm PEG (5 kDa, PEG-4-MI) was purchased from Creative PEGWorks (Durham, NC, USA). 3-mercaptopropioinc acid (MP), 4-mercaptopheylacetic acid (MPA), *p*-toluenesulfonic acid (PTSA), and dithiothreitol (DTT) were purchased from Sigma Aldrich (St. Louis, MO, USA). Tetraethyleneglycol-amine was purchased from BroadPharm (San Diego, CA, USA). Dibenzocyclooctyne acid was purchased from Conju-Probe (San Diego, CA, USA). Fluorescent BSA and fluorescent dextran (70 kDa) were purchased from ThermoFisher (Waltham, MA, USA).

All flash chromatography was performed on a CombiFlash Rf flash column using standard RediSep Rf silica columns (Teledyne ISCO, Lincoln, NE, USA). A Bruker NMR spectrometer (Bruker Daltonics, Billerica, MA) was used to collect ^1^H and ^13^C NMR spectra under standard quantitative conditions (small molecules at 600 MHz with 16 scans for ^1^H and 101 MHz for ^13^C, polymers at 600 MHz with 128 scans for ^1^H). Chemical shifts for protons are reported in parts per million. Data are represented as: chemical shift, multiplicity, coupling constants (Hz), and integration. Mass spectral data from small molecules were collected at a concentration of 0.1 mg/mL using a ThermoFisher Q-Extractive Orbitrap (ThermoFisher Scientific, Waltham, MA, USA).

*Synthesis of thiol end-functionalized four-arm PEG.* Alkyl-thiol (MP) and aryl-thiol (MPA) end-functionalized four-arm PEG were synthesized using previously published methods (Figures S1 and S2) [34]. Briefly, PEG-4-OH (5 kDa, 0.2 mmol, 1 equiv.), MP or MPA (2 mmol, 5 equiv.), and PTSA (0.08 mmol, 0.1 equiv.) were added to an oven-dried round-bottom flask equipped with a Dean–Stark trap and a reflux condenser. Anhydrous toluene (50 mL) was added, and the reaction setup purged with argon at room temperature. The reaction was heated to reflux (120 °C) and stirred for 48 h. Upon completion, the reaction was cooled to room temperature and the PEG precipitated in ethyl ether three times. The polymer was dried overnight under vacuum. The dried polymer was dissolved in anhydrous toluene, added to an argon purged round-bottom flask, and reduced using DTT (1 equiv.) and triethylamine (TEA) (1 equiv.) for 5 h at room temperature. The reaction was acidified with trifluoroacetic acid (TFA) (1.1 equiv.) and the polymer recovered by precipitation in ethyl ether. Subsequently, the polymer was washed an additional time with ethyl ether, dissolved in anhydrous toluene, filtered through a 0.22 µm filter, and precipitated an additional two times in ethyl ether. The resulting polymer was dried under vacuum overnight and the degree of functionalization was assessed using NMR peak integrations. PEG-4-alkyl-SH (functionality = 96%) ^1^H NMR (600 MHz, DMSO-*d_6_*) δ 4.19–4.11 (m, 2H), 3.47 (d, *J* = 5.5 Hz, 113H), 2.75–2.61 (m, 4H), 2.45 (t, *J* = 7.9 Hz, 1H) (Figure S3). PEG-4-aryl-SH (functionality = 93%) ^1^H NMR (600 MHz, deuterium oxide) δ 7.19 (d, *J* = 46.5 Hz, 4H), 5.35 (s, 1H), 4.15 (s, 2H), 3.51 (s, 113H), 3.39 (t, *J* = 4.9 Hz, 1H) (Figure S4).

*Synthesis of nitrobenzyl-azide four-arm PEG.* Nitrobenzyl-azide (NB-azide) was synthesized following a previously published protocol without modification [35,36], with full details available in the Supplementary Materials (Figures S5–S9). NB-azide was conjugated to PEG by first adding to a scintillation vial PEG-4-amine (5 kDa, 0.47 mmol, 1 equiv.) followed by anhydrous dimethylformamide (DMF) (750 µL) (Figure S10). The resulting mixture was vortexed until the PEG was dissolved. Next, nitrobenzyl-azide (NB-azide) (0.42 mmol, 2.2 eq) was added to an oven-dried round-bottom flask and dissolved in anhydrous DMF (750 µL) under argon. Next, 1-[Bis(dimethylamino)methylene]-1H-1,2,3-triazolo[4,5-b]pyridinium 3-oxide hexafluorophosphate (HATU) (0.4 mmol, 2.1 eq) and diisopropylethylamine (DIPEA) (0.97 mmol, 5 eq) were added to the NB-azide mixture. Subsequently, the PEG solution was added to the NB-azide containing solution and the reaction mixture was stirred overnight at room temperature under argon. The next day, the resulting polymer solution was precipitated in ethyl ether, placed at −20 °C for 30 min, and filtered. The product was washed twice more with ethyl ether and dried under vacuum overnight. The dried polymer was dissolved in DI water (5 mL) and dialyzed (Spectra/Por 7 dialysis tubing, 1 kDa MWCO, Repligen) against DI water (2000 mL, 3 changes over 20 h) followed by lyophilization. The functionality of the resulting NB-azide end-functionalized four-arm PEG (PEG-4-NB-azide) was determined using ^1^H NMR peak integrations for the amide (δ = 7.90) and the ring protons (δ = 7.49 and δ = 7.06). PEG-4-NB-Az (functionality = 99%) ^1^H NMR (600 MHz, DMSO-*d_6_*) δ 7.83 (s, 1H), 7.49 (s, 1H), 7.31 (s, 1H), 7.06 (s, 1H), 6.04 (s, 1H), 3.98 (s, 2H), 3.84 (s, 3H), 3.50 (s, 130H), 3.14 (s, 2H), 3.01 (s, 2H), 2.17 (s, 2H), 1.88 (s, 2H), 1.44 (s, 3H), 1.17 (s, 1H) (Figure S11).

*Synthesis of dibenzocyclooctyne four-arm PEG.* Dibenzocyclooctyne (DBCO) end-functionalized four-arm PEG (PEG-4-DBCO) was synthesized following a similar method as outlined above for the synthesis of PEG-4-NB-azide (Figure S12). Briefly, PEG-4-amine (5 kDa, 0.02 mmol) was dissolved in anhydrous DMF (500 µL) in an oven-dried scintillation vial. To a second oven-dried scintillation vial was added DBCO acid (0.18 mmol, 2.2 equiv.), HATU (0.17 mmol, 2.1 equiv.), and anhydrous DMF (500 µL). DIPEA (0.74 mmol, 5 equiv.) was added to the DBCO acid mixture followed by the addition of the PEG solution. The reaction mixture was stirred overnight at room temperature under argon. The resulting polymer solution was precipitated in ethyl ether, place at −20 °C for 30 min, and filtered. The product was washed twice more with ethyl ether and dried overnight under vacuum. The dried polymer was dissolved in DI water (1 mL) and dialyzed (3 mL Slide-A-Lyzer Dialysis Cassette, MWCO 2 kDa, ThermoFisher) against DI water (500 mL, 4 changes over 8 h) followed by lyophilization. The functionality of the resulting PEG-4-DBCO was determined using NMR peak integrations for the ring protons (δ = 7.70–7.26 and δ = 5.04). PEG-4-DBCO (functionality = 89%). ^1^H NMR (600 MHz, DMSO-*d_6_*) δ 8.75–8.35 (m, 3H), 7.96 (s, 1H), 7.73 (t, *J* = 5.7 Hz, 1H), 7.70–7.26 (m, 8H), 5.04 (d, *J* = 14.0 Hz, 1H), 3.77–3.42 (m, 113H), 2.58 (dt, *J* = 16.0, 7.7 Hz, 1H), 2.24 (dt, *J* = 15.4, 7.7 Hz, 1H), 2.00 (m, 1H), 1.79 (dd, *J* = 8.4, 5.9 Hz, 1H) (Figure S13).

*In situ rheologic characterization of bulk hydrogels*. The storage and loss moduli of the hydrogels formed between thiol and maleimide functionalized PEGs were monitored using dynamic time sweep measurements on an AR-G2 rheometer (TA Instruments, Newark, DE, USA). All measurements were conducted with a 1% strain and at a frequency of 2 rad/s, within the linear viscoelastic regime for these hydrogels. The final modulus and the polymerization time were determined to be the point when the storage modulus changed less than 1% between time points. The gel point was less than 30 s for almost all hydrogel formulations, which is less than the time it took to mix the hydrogel forming monomers, load them on the plate, and lower the rheometer top-plate geometry. As such, the first storage modulus value measured by the rheometer was recorded and used to assess the relative rapidness of gelation.

Bulk mechanical properties were determined in situ on 3.33 wt% hydrogels, selected based on preliminary microgel formation experiments and previously reported investigations [34,36], formed in the presence of 5 mg/mL dextran (70 kDa) to mimic microgel formation conditions (e.g., equal parts 5 wt% PEG-4-SH, 5 wt% PEG-4-MI, and PBS containing 15 mg/mL dextran to mimic convergence of three streams). Thiol-Michael hydrogels were formed by reacting 5 µL of 5 wt% PEG-4-MI (0.1× PBS pH = 6), 5 µL 5 wt% PEG-4-MP:MPA (0.1× PBS pH = 5, 9:1 MP:MPA or 1:1 MP:MPA ratio), and 5 µL 5 mg/mL 70 kDa rhodamine-dextran (0.1× PBS pH = 5). The solution was vortexed for 10 s prior to adding 6.5 µL to the bottom Peltier rheometer plate and lowering the upper geometry onto the solution for data collection.

SPAAC hydrogels were characterized similarly to thiol-maleimide hydrogels but using a UV–vis curing accessory as the bottom plate. Briefly, 5 wt% PEG-4-DBCO and 5 wt% PEG-4-NB-Az were mixed at a one to one volumetric ratio with 1× PBS pH 7.4 to make gels with a final polymer concentration of 3.33 wt%. The mixture was pipetted onto the quartz plate of the UV–vis curing agent accessory, the gap lowered, and the gels polymerized for 30 min with mineral oil placed around the outside of the geometry after gelation to prevent hydrogel dehydration. After 30 min, light centered at 365 nm (I_0_ = 2 mW cm^−2^) supplied through an Exfo Omnicure Series 2000 light source was applied through the quartz plate to confirm the degradation behavior of the light-responsive hydrogels was similar to previous reports [9,36].

*Microfluidic device design and fabrication.* Microfluidic devices were fabricated with molded polydimethylsiloxane (PDMS) (Ellsworth Adhesives, Centennial, CO, USA) using soft lithography [37]. Briefly, a silicon wafer (Wafer World, West Palm Beach, FL, USA) was patterned with SU-8 negative photoresist (Kayaku, Westborough, MA, USA) and patterned via UV contact photolithography to create the flow-focusing device master mold (Figure S5). PDMS was mixed at a 10:1 (elastomer to curing agent) ratio, poured over the wafer, and degassed in a vacuum for 20 mins. The PDMS was then cured in a 70 °C oven for 2.5 h. Once cured, devices were cut out and the inlets and outlet were punched with a sharpened 20G blunt syringe tip. Devices were rinsed thoroughly with ethanol and dried with pressurized air before being oxygen plasma treated for 30 s at ~750 mtorr (Harrick Scientific, Ithaca, NY, USA) for bonding to glass slides (Fisher Scientific, Waltham, MA, USA). Glass slides were prepared for bonding by first flaming, then rinsing with ethanol, and oxygen plasma cleaning for five minutes at ~750 mtorr, before treating them with the oxygen plasma for 30 s with the PDMS devices. After bonding, the devices were then heat treated in a 100 °C oven for four hours to accelerate hydrophobicity recovery.

*Formation of microgels using microfluidic devices*. Microgels were formed using the above-described device. The discontinuous, or aqueous phase consisted of two PEG macromers separated by a buffer stream, which were delivered by three separate inlets. The continuous phase consisted of 2 wt% 008-FluoroSurfactant in HFE7500 (Ran Biotechnologies, Beverly, MA, USA). Three aqueous inlets were used to keep the monomer solutions separate until droplet formation to ensure the microfluidic device channels were not clogged due to premature hydrogel gelation. All components were pumped into the microfluidic device through Tygon Microbore tubing (ID = 0.01”, length = 30 cm) attached to 1 mL plastic syringes using 30G blunt needle tips. The polymer components were pumped into the device using a positive displacement double headed syringe pump (Havard Apparatus, Holliston, MA, USA) at a flow rate of 2 µL/min for each stream. The buffer stream containing the desired encapsulant was introduced using a positive displacement syringe pump (New Era Pump Systems Inc., Farmingdale, NY, USA) at a flow rate of 2 mL/min. The total discontinuous phase flow rate was 6 mL/min. The oil stream was pumped into the device using a positive displacement syringe pump (New Era Pump Systems Inc., Farmingdale, NY, USA) at a flow rate of 10 µL/min (same tubing setup as other components). After the flow of all streams stabilized, the flow rate of the aqueous stream was slightly adjusted such that each component accounted for one-third of the channel cross sectional area prior to droplet collection.

For thiol-Michael microgels, 5 wt% PEG-4-alkylSH:arylSH (MP:MPA) was dissolved in 0.1× PBS pH 5, 5wt% PEG-4-alkylSH (MP) was dissolved in 0.1× PBS pH 7, and 5 wt% PEG-4-MI was dissolved in 0.1× PBS pH 6. Dissolved PEG-4-maleimide and BSA or dextran solutions were kept at −20 °C between use and remade after three freeze–thaw cycles. Prior to each use, thiol containing solutions were made fresh from dry, lyophilized powder stored at −20 °C. The center buffer channel was 0.1× PBS pH 7, 0.1× PBS pH 6, or 0.1× PBS pH 5.5 for 0%, 50%, or 90% degradable microgels, respectively. For light-responsive compositions, 5 wt% PEG-4-DBCO and 5 wt% PEG-4-NB-Az were dissolved in 1× PBS pH 7.4, and the center buffer channel was 1× PBS pH 7.4. Dissolved azide and cyclooctyne solutions were kept at −80 °C between use and remade after two freeze–thaw cycles. For determining microgel size, all center PBS channels contained 1 mg/mL BSA-AlexaFlour (AF) 488. For redox-responsive microgels used in degradation studies, 5 mg/mL of 70 kDa TRITC-dextran was incorporated through the middle PBS stream. For light-responsive microgels used in degradation studies, 1 mg/mL BSA-AF488 was incorporated into the microgels.

After formation, microgels were left to polymerize for overnight (~20 h) at room temperature, protected from light, prior to being isolated from the oil phase. Subsequently, microgels were collected using a 10-µm cell filter and washed 5 times with 0.1× PBS (1 mL) to remove excess oil. The washed microgels were collected from the filter and centrifuged (5 min at 4000 revolutions per minute (RPM)). The supernatant was carefully removed from the microgels and replaced with a 70% ethanol solution (5 mL) and incubated at room temperature for 5 min followed by centrifugation to sediment microgels. The ethanol solution was removed and replaced with 0.1× PBS pH 7 (5 mL), incubated for 5 min, and centrifuged. The microgels were then dried overnight at room temperature under vacuum in a preweighed microcentrifuge tube. The resulting dried microgels were weighed and resuspended at 4 mg/mL prior to use in experiments. To determine the size of the microgels, the microgels were resuspended in 1× PBS pH 7.4 and imaged using a confocal laser scanning microscope (Zeiss LSM 800; laser excitation at 488 nm). Particle counts and particle diameter were determined using ImageJ’s Analyze Particles function.

*Degradation of thiol-maleimide microgels*. Dried redox-responsive microgels containing either 50% or 90% degradable content and formed with 5 mg/mL TRITC-dextran were re-suspended in DI water at 4 mg/mL, centrifuged (4000 RPM) for 5 min, decanted, and resuspended in fresh 1× PBS pH 7.4. Microgels then were swollen overnight in PBS to reach an equilibrium swollen state prior to degradation experiments. The swollen microgels were centrifuged (4000 RPM) for 5 min to remove the PBS and subsequently resuspended to 1.3 mg/mL in 0.1 mM GSH (low reducing), 10 mM GSH (high reducing), or 1× PBS pH = 7.4 (normal hydrolysis). Prior to each experiment, GSH solutions were made fresh from dry, lyophilized powder stored at 4 °C. The microgel suspension (200 µL) was transferred to a 96-well black plate with a clear bottom (iBidi, Fitchburg, WI, USA) and imaged on a confocal LSM (Zeiss LSM 800; laser excitation at 561 nm) at predefined time points over 16 days. Buffer exchanges were conducted every two days and degradation was performed at 37 °C at 5% CO_2_ to mimic in vivo conditions. For buffer exchanges, the plate containing particles was centrifuged (2000 RPM) for 2 min to settle microgels to the bottom of the well followed by the careful exchange of half the degradation buffer (100 µL). Z-projections of confocal stacks (120 µm-thick with 10 µm-slice intervals) were made, and then particle counts and particle areas were determined using ImageJ’s ‘Analyze Particles’ function, which was set to analyze circles (range 60–150 µm in diameter), and used to monitor the degradation process.

*Degradation of light-responsive SPAAC microgels*. Dried light-responsive microgels formed using 1 mg/mL BSA-AF488 were re-suspended in DI water at 4 mg/mL, centrifuged (4000 RPM) for 5 min, decanted, and resuspended in fresh 1× PBS pH 7.4. Microgels were then swollen overnight, centrifuged, and the PBS replaced with fresh PBS. The photodegradable hydrogels were loaded into a 96-well black plate with a clear bottom and imaged on a confocal LSM (Zeiss LSM 800; laser excitation at 488 nm). After initial imaging, the microgels were subjected to flood irradiation using the light source on the microscope itself: light was supplied by the epifluorescent light source that was passed through the DAPI filter (Mercury HBO; Carl Zeiss 380301-9350-000; I_0_ = ~1.5 mW cm^−2^ measured at 365 nm; excitation output ~300–400 nm, Zeiss FL Filter Set 49) of the microscope with a 10× objective. This approach of using the light from the microscope for sample irradiation was selected for its accessibility, given the broad use of and access to fluorescent microscopes across laboratories in different disciplines, as well as its allowing the plate to remain undisturbed during imaging and irradiation steps. The plate remained unmoved during the irradiation process and the microgels were immediately imaged again following the 30 s of irradiation. Next, targeted degradation of individual microgels was performed by exposure of single microgels to focused light supplied through the 40× objective for 10 s, where only single particles within the field of view were irradiated by closing down the microscope aperture for illuminating only that portion of the field of view during irradiation. Microgels were imaged through a 20× objective prior to and after targeted light exposure with the same parameters utilized for imaging microgel degraded by flood irradiation.

*Statistics*. All statistics represent the mean plus or minus the standard error of the mean with n = 3 independent samples unless otherwise stated. One-way ANOVA with post hoc Tukey’s Honest Significant Difference (HSD) was performed to compare mechanical properties, microgel diameters, and release behavior between the different conditions. Coefficients of variation, a standardized measure of the dispersion of a distribution, were calculated for the microgel populations produced with different formulations by dividing the standard deviation by the mean.

## 3. Results and Discussion

### 3.1. Bulk Hydrogel Formation and Mechanical Properties

Two different spontaneous click chemistries were used to form hydrogels (thiol-Michael and SPAAC) for subsequent translation to microgels, and all hydrogels were formed using end-functionalized four-arm PEG. PEG-based macromers were selected for their ease of modification, lack of protein interaction, and biocompatibility. Degradable crosslinks were formed within thiol-Michael hydrogels by controlling the ratio of thioether succinimide bonds containing alkyl-SHs to those containing aryl-SHs, as aryl-SH-based crosslinks undergo a retro-Michael addition that can lead to network degradation in the presence of exogenous thiols (e.g., GSH that is found in increased concentrations in many tumor types) [34,38]. For GSH-responsive gels, conditions containing 90% and 50% redox-degradable content (i.e., aryl-SH content with balance of non-degradable alkyl-SH content) were selected for their desirable formation characteristics balanced with their ability to degrade on relevant time scales (on the order of days to weeks), as detailed below [9]. In addition to the GSH-responsive gels, we wanted to demonstrate the utility of the system by forming microgels with different polymerization and degradation mechanisms. These hydrogels were formed using a SPAAC reaction, where nitrobenzyl (NB) crosslinks were incorporated into the backbone of the hydrogel network to impart light-responsiveness and provide on-demand control over protein release. The use of spontaneous click chemistries in the formation of microgels using MFF has been limited due to their rapidness of gelation, which can lead to challenges in MFF device operation [19,39]. Notably, to date, redox-responsive thiol-Michael and light-responsive SPAAC chemistries have not been integrated into microgels formed using MFF.

The mechanical properties of bulk hydrogels representing the various formulations to be used in microgel formation were determined using in situ rheometry (Figure S15). Of note, when investigating the mechanical properties, was controlling the gelation time of the formulations such that they could be integrated within MFF to form microgels. All thiol-Michael gels were formulated using reduced buffer strength (0.1× PBS), decreased pH (thiols in pH 5 or 6), and varied ratios of alkyl to aryl thiols for the purposes of consistent microgel formation, building upon approaches identified within the literature [40,41,42]. In preliminary experiments, it was observed that hydrogels with 100% aryl-thiol crosslinks gelled almost instantaneously (<10 s) when formed using either regular or reduced strength PBS even at decreased pH. To counter this, we added non-degradable alkyl-thiol content to the hydrogels as the higher pKa of the alkyl-thiol aids in slowing the gelation rate [42]. This strategy along with using reduced strength PBS at decreased pH, inspired by literature reports [40,41], allowed gelation of thiol-Michael hydrogels with both aryl and alkyl thiols on the order of 30 s or greater, where 30 s was found to be the minimum time to gelation needed for consistent microgel formation using the MFF device. The resulting 90% and 50% redox-responsive formulations were selected for further investigation due to their favorable gelation kinetics and their ability to fully degrade in response to GSH stimulus, where a minimum of 50% of the crosslinks within the hydrogel backbone need to be degradable for complete degradation to be achieved based on Flory Stockmayer theory [43]. These adjustments to pH, buffer strength, and thiol pKa were used to address the rapid gelation and subsequent viscosity changes presented by thiol-Michael reactions, especially those utilizing thiols with a high pKa (e.g., aryl-thiols), that can lead to inconsistent formation of microgels using the MFF device. SPAAC gels were formed by reaction of macromers functionalized with DBCO and azide, respectively, which form hydrogels on a similar time scale.

**Figure 2:**
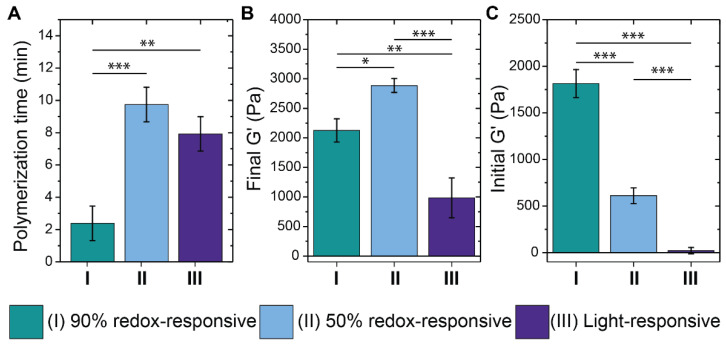
*Mechanical properties of hydrogel formulations.* The polymerization time (**A**), the final in situ storage modulus (**B**), and the initial in situ storage modulus value recorded by the rheometer (**C**) were examined for each hydrogel formulation. The final formulations investigated were (I) 90% and (II) 50% redox-sensitive crosslinked and (III) NB light-responsive SPAAC crosslinked hydrogels. Shown is the average and standard error for n = 3 samples, where * indicates *p* < 0.05; ** indicates *p* < 0.01; *** indicates *p* < 0.001.

Differences were observed among the final in situ bulk hydrogel storage moduli for the three different formulations (Figure 2A). For the GSH-responsive hydrogels, the difference in final modulus was inversely related to the initial storage modulus, which is the modulus observed for the first measurement taken for each sample during in situ formation. While the formulations gelled too quickly to capture the gel point with rheometry, one can think of the initial modulus during in situ formation as a rough surrogate for the gel point, where faster gelation leads to a higher initial modulus during in situ measurements of hydrogel formation. Hydrogels formed with 50% redox-degradable content had a lower initial modulus and a higher final modulus than those containing 90% degradable content. These observations of higher initial modulus and lower final modulus for the 90% degradable formulation suggested rapid initial polymerization that may have been followed by diffusion limitations at later times in the network formation, introducing defects (i.e., limiting the formation of elastically active crosslinks) [43,44,45] (Figure 2B). The observation of increased polymerization time for 50% redox-degradable hydrogels further supports their slower yet continued reaction, leading to a higher modulus (Figure 2C). These observations are consistent with the literature: the enhanced diffusivity of reactive macromers through the network prior to being ‘trapped’ has been shown previously to lead to decreased heterogeneity in the network structure and thus a higher final modulus [44,45]. Interestingly, the final modulus of hydrogels formed using SPAAC chemistry was much lower than the thiol-Michael conditions, despite having the same theoretical network structure based on macromer structure (length and number of arms), and SPAAC hydrogels exhibited low initial modulus and longer polymerization time during in situ formation. This difference in modulus may be due to SPAAC hydrogel formulations being slightly off-stoichiometry owing to slight differences in functionalization between the azide and cyclooctyne PEG components.

Taken together, these observations highlight some of the differences in mechanical properties one encounters when changing linker chemistry. Overall, these reactive chemistries provide opportunities for different levels of network structure control initially, over time (e.g., in response to stimuli), and orthogonally (e.g., to other reactive handles or biological molecules). Even with the slight differences in modulus that were observed, the different thiol-Michael and SPAAC gels all formed consistently and on relevant time scales for microgel formation, and these formulations were utilized in all subsequent experiments to demonstrate utility of the MFF microgel formation.

### 3.2. Formation of Thiol-Michael PEG Microgels Using Flow Focusing Microfluidics

**Figure 3:**
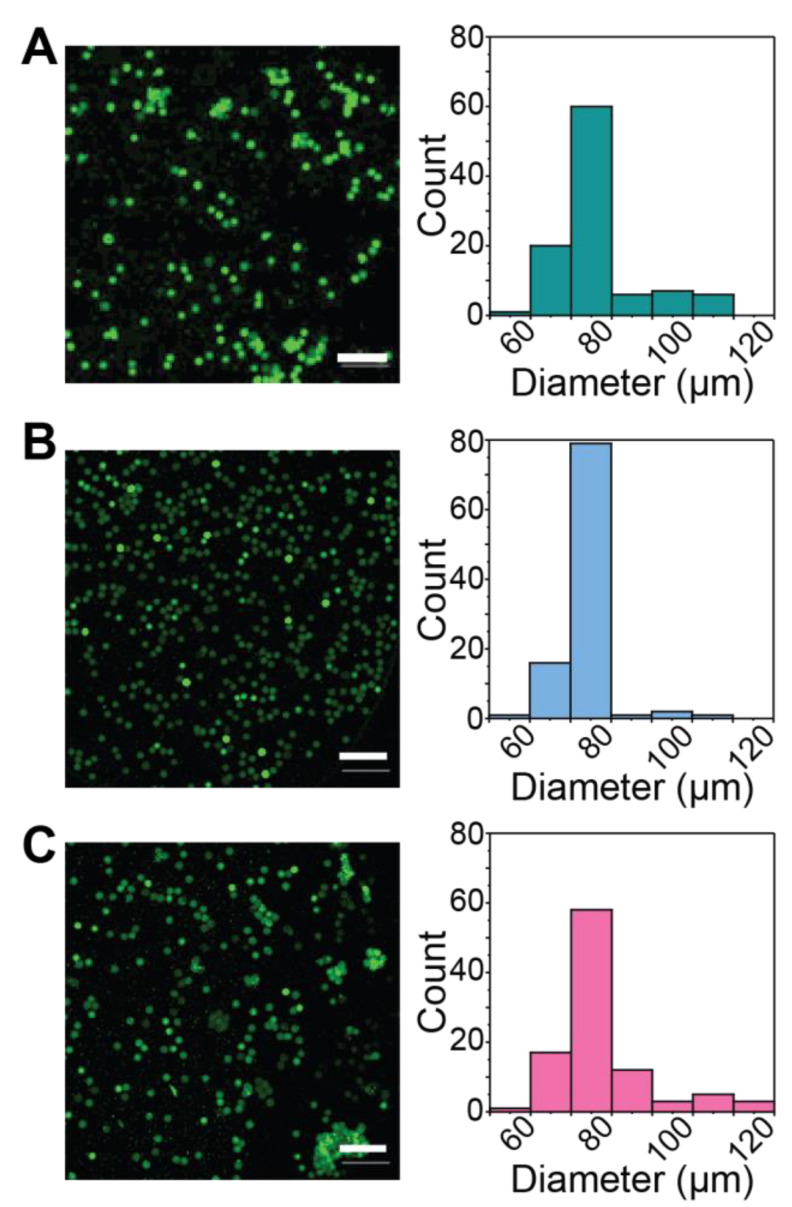
*Formation of redox-responsive microgels.* Microgels were formed by introducing PEG-4-arylSH:alkylSH and PEG-4-MI precursor components through opposite inlets with a buffer stream containing the desired protein of interest for encapsulation (BSA-AF488 here): homogeneous microgels were formed that contained (**A**) 90% (average diameter = 77.9 ± 12 µm), (**B**) 50% (average diameter = 73.9 ± 6 µm), or (**C**) 0% (average diameter = 76.7 ± 10 µm) degradable content. Scale bars = 500 µm. Representative images are shown on the left with histograms showing the distribution of diameters on the right.

MFF of immiscible fluids provides a route to form homogeneous microgel populations, which have been utilized for applications ranging from drug delivery to cell encapsulation and tissue engineering [14,46,47]. When utilized for spontaneous click chemistries, it is important to maintain component separation until immediately before the droplet is formed to prevent clogging of the device due to premature hydrogel formation. To this end, three aqueous inlet channels were merged just prior to the combined stream being pinched off by a fluorinated oil stream to form a droplet (Figure 1A). Additionally, we found, through early experiments, that the viscosity and polymer concentration of the macromer containing streams needed to be similar for consistent microgel formation to be achieved. Within this device, the 5 wt% thiol end-functionalized four-arm PEG (different ratios of PEG-4-alkyl-SH:aryl-SH) and 5 wt% maleimide end-functionalized four-arm PEG (PEG-4-MI) were introduced through the outside channels and buffered by a PBS stream containing a model protein cargo (bovine serum albumin (BSA)-488), which is similar in size to many bioactive growth factors and cytokines and allows easy visualization of the resulting microgels and protein release [48]. Microgels containing 90%, 50%, and 0% redox-degradable content were successfully formed and observed to have similar diameters, showing that alterations to the degradable content and differences in the crosslinking kinetics observed for 90% and 50% aryl-thiol conditions did not alter microgel formation properties (Figure 3). In addition, all formulations had low coefficients of variation (CV_90%_ = 15%; CV_50%_ = 8%; CV_0%_ = 13%), indicating homogenous microgels especially when compared to batch emulsion or extrusion fragmentation techniques that typically result in higher levels of dispersity (e.g., CV ~30–40% for batch emulsions [15]; preliminary findings with inverse suspension polymerization resulted in CV ~70% (Figure S16)). The dispersity of particles formed here was within the range of that observed for other PEG-based microgels formed using microfluidic flow-focusing methods (CV = 5–20%) [19,49].

Further, all microgel formulations were found to have an average diameter of ~ 80 µm, indicating that they could be passed through standard needles used for injections. Indeed, microgels were successfully passed through a syringe with a small diameter needle (30G, well within size range recommended for injections clinically [50]) without observable damage to microgels (Figure S17). The size of the microgels also should allow them to remain localized within tissues at the injection site, as they are too large to be up taken by macrophages or similar cells or to circulate freely upon subcutaneous or intramuscular injection [22]. This ease of injection and minimal movement of the microgels from the injection site make them good candidates for controlled localized protein release. Taken together, these studies demonstrate operating conditions and a MFF device design for the formation microgels with rapid click chemistries and relevant for the encapsulation of biocargoes, expanding the arsenal of tools available for microgel generation and with complementarity to other techniques (e.g., suspension polymerization and bulk hydrogel fragmentation) [10,15].

### 3.3. Degradation of Redox-Sensitive Microgels

**Figure 4:**
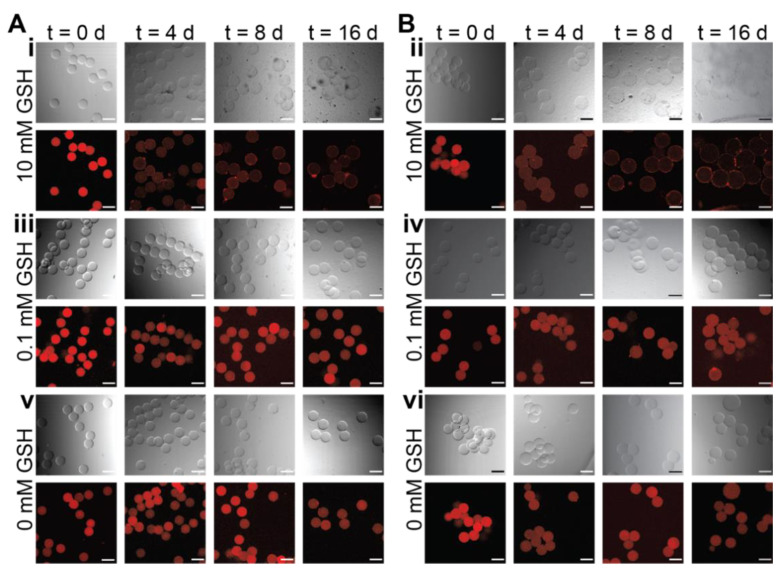
*Degradation of microgels containing redox-sensitive crosslinks*. Microgels containing 50% (**A**) or 90% (**B**) redox-sensitive crosslinks and 5 mg/mL 70 kDa dextran-TRITC were incubated in either 10 mM GSH (**i**,**ii**), 0.1 mM GSH (**iii**,**iv**), or PBS pH = 7.4 (**v**,**vi**). Representative brightfield (top) and fluorescent (bottom) images of the microgels at 0, 4, 8, and 16 days are shown. Scale bars = 50 µm.

Microgels formed with either 50% or 90% redox-degradable content and loaded with fluorescent biopolymer cargo (tetramethylrhodamine (TRITC)-dextran) were degraded by incubation in either 0.1 mM GSH (~normal healthy tissue concentration), 10 mM GSH (~intracellular concentration), or PBS pH = 7 (Figure 4). Here, 70 kDa dextran was utilized for these demonstrations as it has been shown to have similar encapsulation and release behavior as IgG-1 type antibodies with PEG-based hydrogels [51]. Microgels were imaged and the degradation buffer exchanged with fresh buffer every two days. Representative images of hydrogels containing 50% redox-degradable linkages show that microgels incubated in 10 mM GSH begin to degrade after 4 days with almost full degradation observed after 16 days (Figure 4A(i)). For microgels containing 90% redox-degradable content and incubated in 10 mM GSH, degradation began after 2 days with almost full degradation observed after 8 days (Figure 4B(ii)). While ‘outlines’ of the microgels incubated in 10 mM GSH can be seen on the surface of the well plate after sixteen days, upon careful removal of the buffer, no microgels were observed on the plate surface for these conditions, suggesting their complete degradation and dissolution; notably, microgels were still observed in the lower reducing environment conditions. Based on these observations together with quantitative analysis on the microgels below, we hypothesize that the microgels in the high reducing environments were degraded at this late time point but the lack of mixing and centrifugation of the plates prior to imaging led to deposition of the dextran along the plate surface. As expected, minimal degradation for both microgel formulations incubated in either 0.1 mM GSH or PBS pH = 7.4 was observed over the experimental timeframe (Figure 4) [34,42].

**Figure 5:**
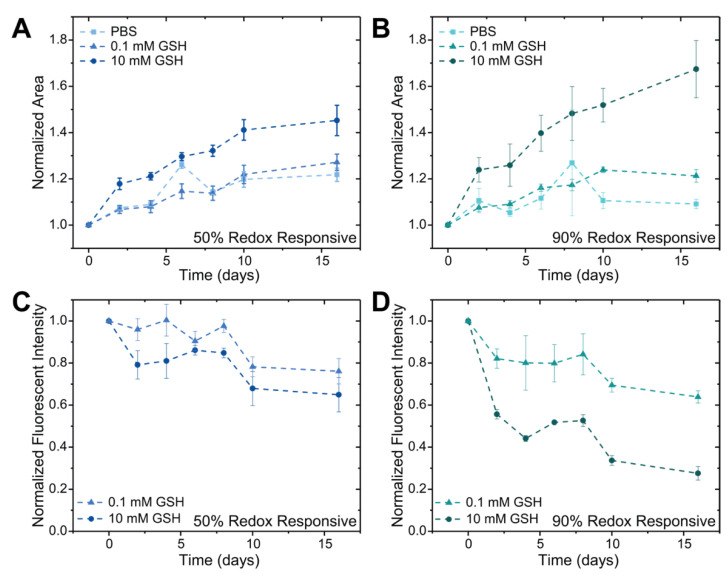
*Characterization of microgels degraded in aqueous or reducing conditions*. The area of the microgels, determined using the circular area of microgel confocal projections, at each time point was determined for microgels containing either 50% (**A**) or 90% (**B**) redox-degradable crosslinks for a period of 16 days as a measure of microgel degradation. To assess release of dextran-TRITC from the degraded 50% (**C**) and 90% (**D**) formulations, the fluorescent intensity inside the microgels was determined and normalized to microgels incubated in PBS to account for potential photobleaching of the TRITC group over time.

Either microgel area or volume can be used to assess the swelling ratio of the hydrogel network, which is related to the crosslink density and mesh size of the network [52]. Accordingly, microgel area, based on circle confocal projection, was measured to more quantitively compare the rates of microgel degradation between the two populations. By observing microgel projected area, we again observe that microgels containing 90% redox-degradable crosslinks degraded faster than those containing 50% redox-degradable crosslinks, which was anticipated based upon the ratio of degradable to non-degradable content (Figure 5A,B). Interestingly, the microgel formulations investigated degraded slower than reports for similar bulk hydrogel formulations [53]. We hypothesize that this is due to increased ring-opening in the microgels that occurs during the overnight incubation of the microgels in the polymerization buffers used to ensure stable microgel formation; here, ring-opening refers to the phenomenon that occurs when the thioether succinimide bond formed upon reacting a thiol and maleimide ring-opens, causing the bond to no longer be able to undergo a retro-Michael addition for network degradation [42,53]. This increased time at decreased pH compared to that of bulk hydrogels, which are washed with fresh buffer ~1 h after formation compared to after ~20 h, would likely increase the rate of ring opening as it has been reported that changes to the solution pH can be used to increase the rate of ring-opening to create stable thioether succinimide linkages [42,51].

In addition to observing the change in the microgel area over time, the release of dextran was monitored over the experimental timeframe by determining the average fluorescent intensity within the microgels. The fluorescent intensity measured at each time point was first normalized to the average intensity of control PBS microgels at that time point, to account for any possible photobleaching caused by imaging, followed by normalization to the fluorescent intensity of that condition at t = 0, to account for any possible variations in dextran loading. Within the 50% redox-degradable formulation, decreases in the average fluorescent intensity were observed to be similar for formulations incubated in both 0.1 and 10 mM GSH, likely due to the rate of degradation of the hydrogel network being on the same time scale as hindered diffusion of the dextran out of the hydrogel network (Figure 5C). In comparison, differences in the release profile of the 90% redox-degradable microgels incubated in 10 mM GSH versus 0.1 mM GSH were observed (Figure 5D). The rate of degradation of microgels containing 90% redox-sensitive crosslinks in high GSH concentrations was observed to be greater than that of hindered diffusion of the dextran out of the network. Indeed, as the fluorescent intensity for microgels in 10 mM GSH is normalized to that of microgel incubated in 0 mM GSH (control), a flat straight line would be observed for fluorescence intensity if there was no difference in release for the 10 mM condition compared to the 0 mM condition. Consequently, the observed decreases in normalized fluorescence intensity for the microgels in the 10 mM condition supports the importance of microgel degradation in cargo release relative to 0 mM condition, where hindered diffusion of the dextran out of the network would dominate the release mechanism. Further, increased release rates relative to 0 mM control were observed for both microgels containing 50% redox-sensitive crosslinks and those containing 90% redox-sensitive crosslinks incubated in less reductive environments. These results indicate that the increase in microgel size over time, which is known to correlate with the responsive degradation of crosslinks within the polymer network that comprises the microgel, leads to an increase in the mesh size of the hydrogel network and thus enhanced release compared to those with less increases in area [52]. Note, while the modulus of the 50% formulation was higher than the 90% formulation based on in situ rheometric measurements of the bulk hydrogels, no significant differences were observed in the rate of dextran release between these formulations at either no or low GSH concentrations; this observation indicates similar rates of release by hindered diffusion for the base formulations and further supports the importance of linker cleavage for the increased rates of release observed from the 90% formulation in high GSH concentrations. Overall, within microgels populations, both the percentage of degradable content and the amount of GSH in the microenvironment can be utilized to control the release of biopolymers.

### 3.4. Formation and Degradation of Light-Sensitive Microgels

**Figure 6:**
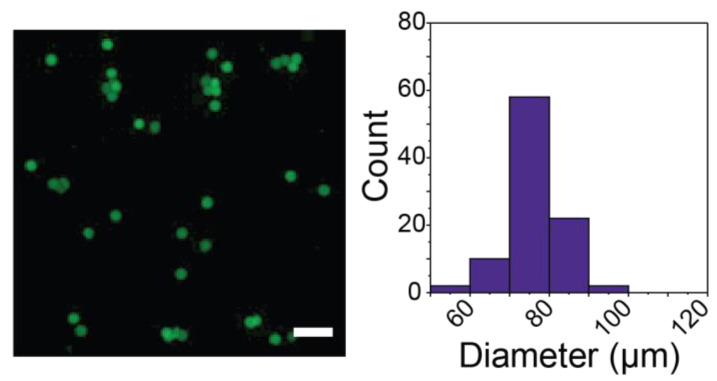
Formation of light-degradable microgels using SPAAC. Homogeneous photodegradable microgels were formed between PEG-4-DBCO and PEG-4-NB-azide and loaded with BSA-AF488 using MFF. Average microgel diameter formed using SPAAC with light-responsive crosslinks was 76.7 ± 6.2 µm. Scale bar = 200 µm. Representative images are shown on the left with histograms showing the distribution of diameters on the right.

Light-triggered degradable formulations for protein release are valuable for creating on-demand release profiles, providing opportunities to tailor release to fit specified treatment regimens by externally controlled burst protein release (Figure 1B). To establish a microgel formulation relevant for exogenously triggered degradation and immediate cargo release with light, a nitrobenzyl light-responsive moiety was incorporated into the hydrogel backbone. Further, to demonstrate the broad utility of the microfluidic system used above, microgels were formed with MFF using SPAAC, a different and complementary spontaneous click chemistry to the thiol-Michael reaction used above. The microgels were formed with 5 wt% PEG-4-DBCO, 5 wt% PEG-4-NB-azide, and BSA-488 used as encapsulated cargo. The resulting SPAAC-formed microgels were not significantly different in size or coefficient of variation (CV = 8%) compared to those formed using the thiol-Michael addition, indicating that this formulation could also be used for localized protein delivery mechanisms (Figure 6). Together, these data demonstrate the utility of the microfluidic device design and formation configurations in creating homogeneous microgels across varying spontaneous click chemistry polymerization mechanisms.

**Figure 7:**
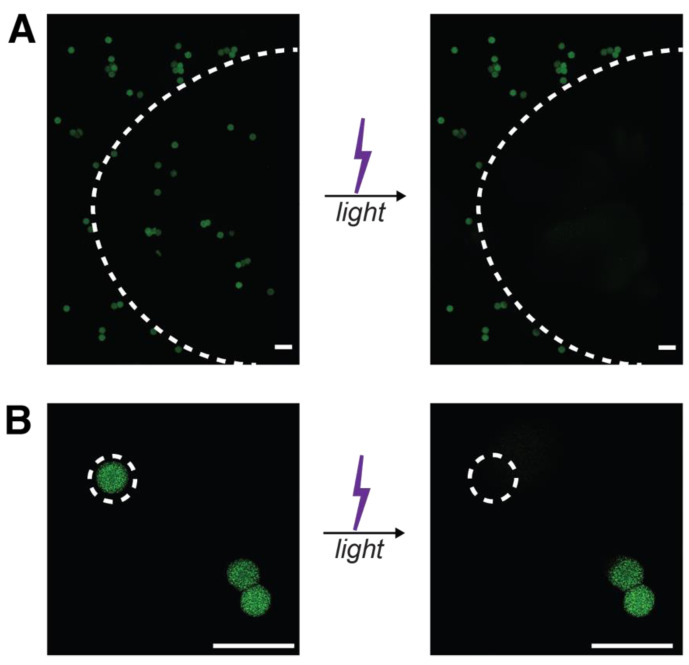
*Degradation of light-responsive microgels*. Light-responsive microgels loaded with BSA-AF488 were degraded by either flood (**A**) or targeted (**B**) long-wavelength UV light irradiation (λ = 300–400 nm; I_0_ = 1.5 mW cm^−2^; t_flood_ = 30 s; t_target_ = 10 s). Areas of light exposure are shown by dashed lines, where irradiation was performed through a 20× objective for flood irradiation and 40× objective with a partly closed aperture for targeted irradiation, and then imaging of the wider field of view was performed with a 10× or 20× objective, respectively. Scale bars = 200 µm.

After SPAAC microgel formation and post processing, light-responsive microgels were resuspended in PBS and swollen overnight prior to being exposed to focused light supplied through the DAPI filter (excitation output ~300–400 nm) of the epifluorescent light source on a confocal LSM. The degradation of the microgels was observed to be rapid under the conditions investigated, highlighting their potential value in being used for burst protein release. Degradation of all microgels within a field of view, irradiated through 20× objective and imaged through a 10× objective, occurred with 30 s of flood irradiation with UV light (I_0_ = 1.5 mW cm^−2^) (Figure 7A), a cytocompatible dose of relevance for degradation of microgels through skin [35]. Next, targeted degradation of the light-responsive hydrogels was achieved by focusing the light on a single microgel, irradiated for 10 s (I_0_ = 1.5 mW cm^−2^) through the 40× objective with a partly closed aperture and imaged through a 20× objective (Figure 7B). The rapid degradation of the microgels, under 10 s, can be attributed in part to the short path length (~80 µm) the light must travel, making the microgels optically thin so that bulk degradation and complete erosion of the microgels are achieved for triggered burst protein release, in addition to the known cleavage kinetics for this photolabile moiety and low polymer weight percent used in microgel formation [36]. The half-life of 120 µm NB hydrogel films degraded on the rheometer with 365 nm alone has been found to be ~3 min, which is similar to the time of irradiation here, supporting rapid degradation of the microgels under this light dosage [36]. Additionally, the nitrobenzyl moiety utilized here has been shown to absorb light strongly within the 300–400 nm range, indicating that degradation would be faster here than observed when degraded with 365 nm light alone for a PEG-based microgel of a similar formulation [54].

Large-scale degradation of the microgels (e.g., with flood irradiation) is of particular interest for on-demand protein delivery applications, whereas targeted degradation (e.g., with focused light) is of more interest for in vitro cell culture applications for the controlled presentation of biochemical cues. In both cases, rapid degradation of the hydrogel network, leading to burst protein release, is particularly desirable to minimize light exposure to tissues or cells while providing a ‘pulse’ of released protein. Local burst release provides a bolus protein dose for modulating local cellular activities over short time scales (i.e., minutes/hours or a few days for growth factors or antibodies against them, respectively). For example, such pulses of released protein may prove useful in wound healing applications, where delivery of growth factor-neutralizing antibodies (e.g., against transforming growth factor beta 1) has been shown to be useful in reducing hypertrophic scarring when delivered between days ~8 and 12 post-wounding of skin within rabbit models [55,56,57].

More broadly, the externally triggered pulsatile release achieved with the light-responsive microgel formulation can be complemented by other release modalities, such as the redox-responsive microgel formulation shown above, that provide sustained protein release (Figure 1B) as desired for future applications of interest. Taken together, the light-responsive and redox-responsive microgels established here highlight how a combination of bottom-up and top-down designs, with careful selection of formation and degradation chemistries and use of MFF, can be utilized to create microgels with various release modulates, from light-triggered burst release to responsive sustained release, for achieving a range of release profiles needed for therapeutic efficacy in different applications. For example, in future applications, one can envision opportunities for loading of the different microgel formulations with the same therapeutic cargo or two different cargoes for achieving desired temporal dosing of a single therapeutic or achieving temporally regulated combination therapies, respectively.

## 4. Conclusions

Innovative redox- and light-responsive microgels loaded with biopolymer cargoes were fabricated using MFF. Redox-responsive microgels were formed using varying ratios of PEG-4-arylSH to PEG-4-alkylSH and found to have consistent diameters independent of formulation, highlighting the benefit of using microfluidic techniques for forming homogeneous microgel populations. After formation, the degradation properties of redox-responsive microgels containing either 90% or 50% redox-degradable content were demonstrated in different reducing microenvironments, which correlated with previous observations of responsive degradation of bulk hydrogels and led to sustained cargo release. Further, light-degradable microgels were formed using SPAAC, which were determined to have a similar diameter to those formed using a thiol-Michael addition. These light-responsive particles rapidly degraded through either flood or targeted light irradiation, leading to burst cargo release. The successful synthesis and degradation of homogeneous populations of innovative redox- and light-responsive microgels using spontaneous click chemistries within MFF devices afford opportunities for creating controlled combination therapy regimens with either sustained or burst protein release. The combination of microgels with sustained release and those with on-demand release can provide opportunities to create complex protein release profiles that have yet to be achieved in current microgel formulations.

## Data Availability

The data presented in this study are available on request from the corresponding author.

## Supplementary Materials

The following supporting information can be downloaded at: https://www.mdpi.com/article/10.3390/pharmaceutics14051062/s1, Figure S1: Scheme for synthesis of PEG-4-alkylSH; Figure S2: Scheme for synthesis of PEG-4-arylSH; Figure S3: ^1^H NMR PEG-4-alkyl-thiol; Figure S4: ^1^H NMR PEG-4-and aryl-thiol; Figure S5–S8: Scheme for synthesis of nitrobenzyl-azide; Figure S9: ^1^H NMR of NB-azide; Figure S10: Scheme for synthesis of PEG-4-NB-azide; Figure S11: ^1^H NMR PEG-4-NB-azide; Figure S12: Scheme for synthesis of PEG-4-DBCO; Figure S13: ^1^H NMR PEG-4-DBCO; Figure S14: MFF device design; Figure S15: Representative rheology time sweep plots; Figure S16: Microgels formed by inverse suspension polymerization; Figure S17: Microgels injected through a needle.
