# Supplementary material for: Microgels Formed by Spontaneous Click Chemistries Utilizing Microfluidic Flow Focusing for Cargo Release in Response to Endogenous or Exogenous Stimuli
Source: Pharmaceutics. 2022 May 15;14(5):1062. doi: 10.3390/pharmaceutics14051062 (PMC9145542; doi:10.3390/pharmaceutics14051062)
Supplement: Supplementary file 1 [file pharmaceutics-14-01062-s001.zip › pharmaceutics-1654964-SI.pdf]

Microgels formed by spontaneous click chemistries utilizing microfluidic flow focusing for cargo release in response to endogenous or exogenous stimuli

Paige J. LeValley,[1] Amanda L. Parsons,[2] Bryan P. Sutherland,[1] Kristi L. Kiick,[3,4] John S. Oakey,[2] and April M. Kloxin[1,3]

[1]Chemical and Biomolecular Engineering, University of Delaware, Newark, DE
[2]Chemical Engineering, University of Wyoming, Laramie, WY
[3]Material Science and Engineering, University of Delaware, Newark, DE
[4]Biomedical Engineering, University of Delaware, Newark, DE

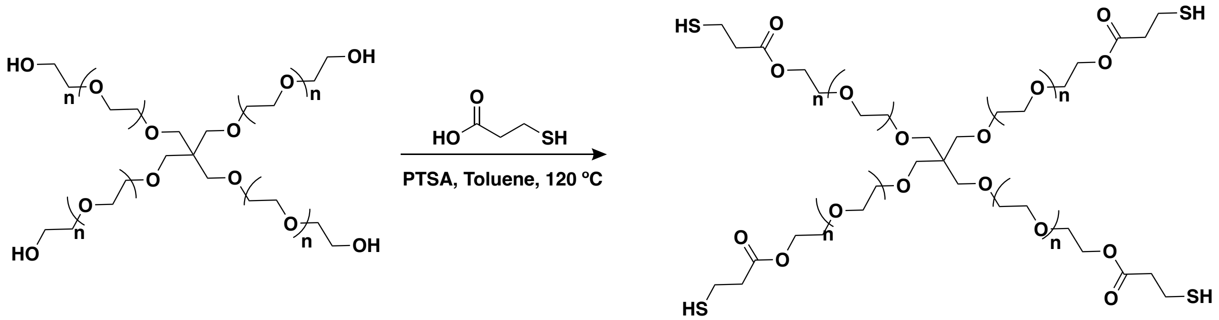

Figure S1. Scheme for the synthesis of PEG-4-alkyl-thiol.

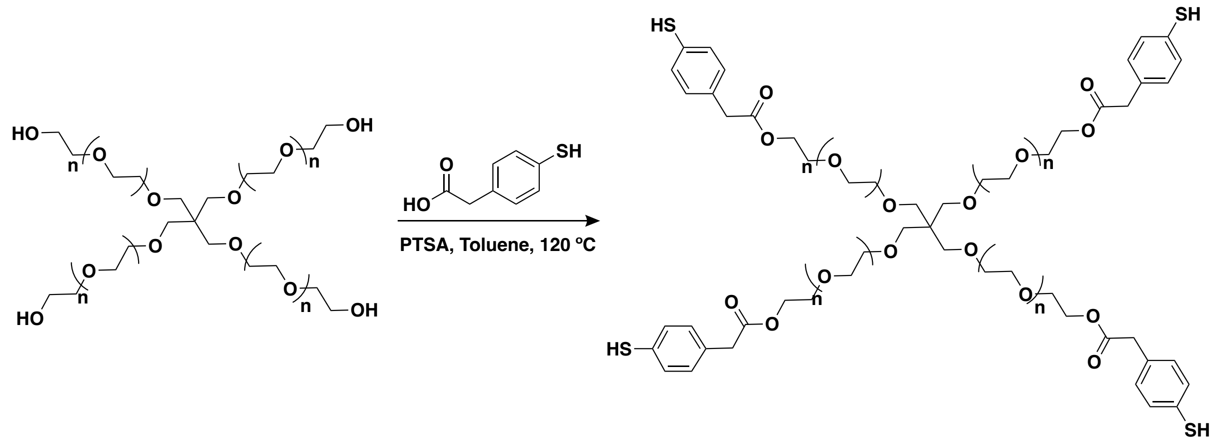

Figure S2. Scheme for synthesis for PEG-4-aryl-thiol.

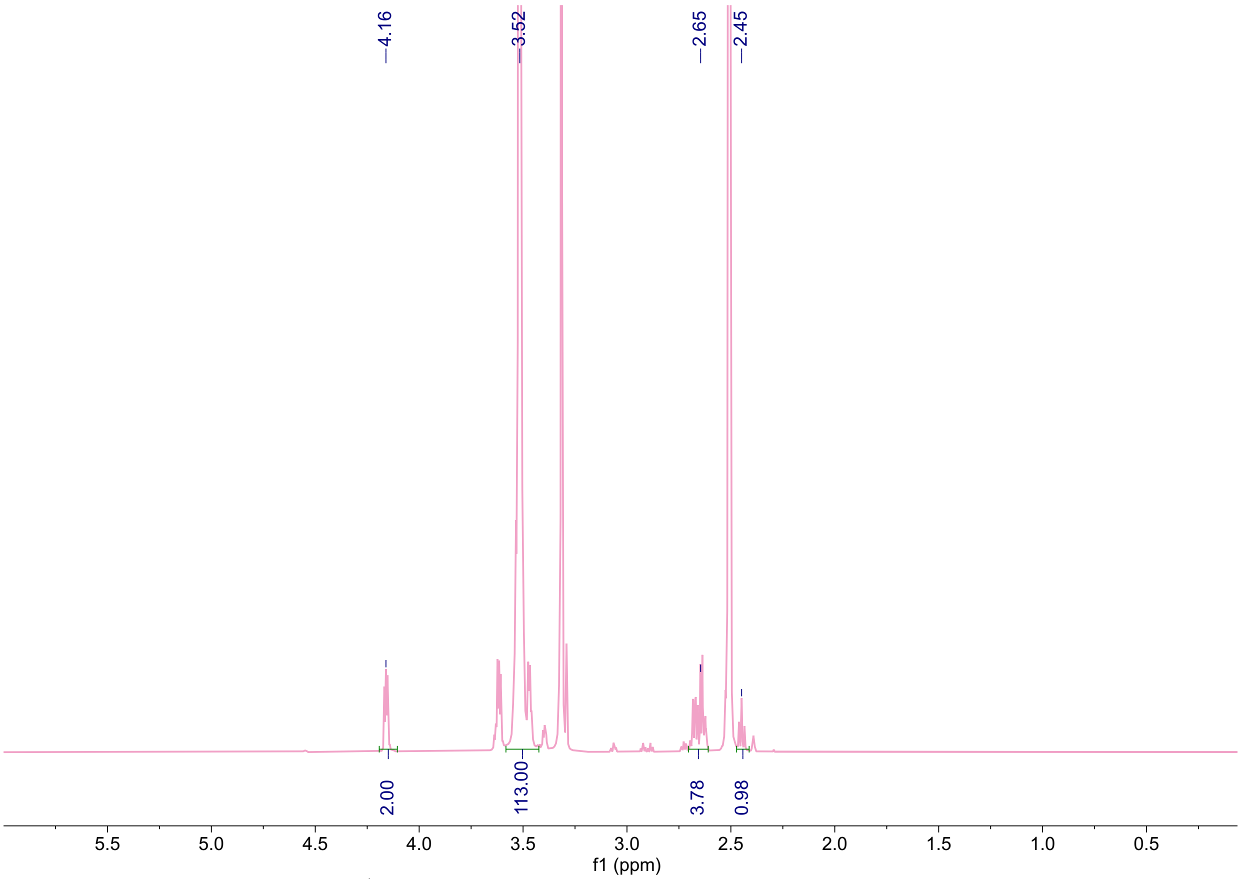

Figure S3. Representative ^{1}H NMR spectrum for PEG-4-alkyl-thiol (600 MHz, DMSO-d_6, 128 scan) δ 4.19 – 4.11 (m, 2H), 3.47 (d, *J* = 5.5 Hz, 113H), 2.75 – 2.61 (m, 4H), 2.45 (t, *J* = 7.9 Hz, 1H). Functionality = 96% based on proton shifts at 2.65 and 2.45.

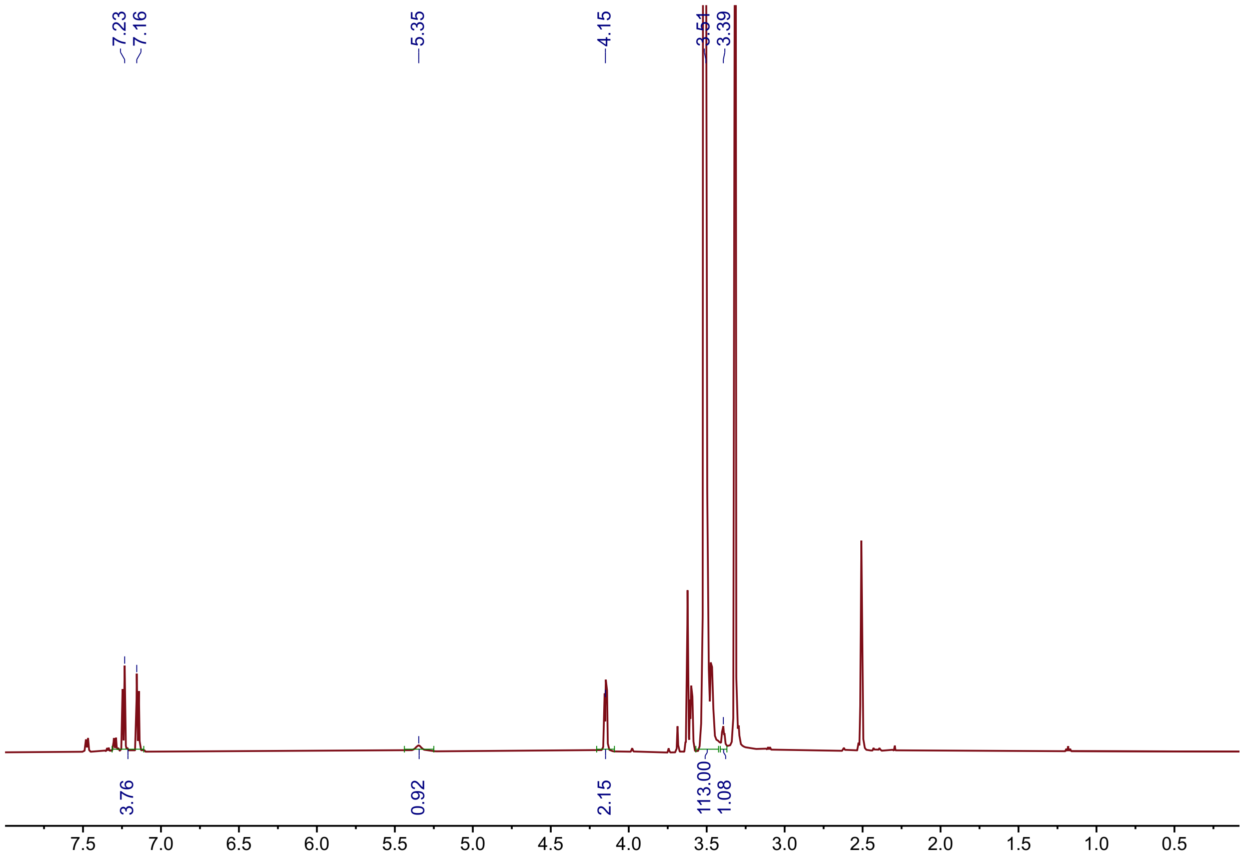

Figure S4. Representative ^{1}H NMR spectrum for PEG-4-aryl-thiol (600 MHz, D_2O-d_6, 128 scan) δ 7.19 (d, J = 46.5 Hz, 4H), 5.35 (s, 1H), 4.15 (s, 2H), 3.51 (s, 113H), 3.39 (t, J = 4.9 Hz, 1H). Functionality = 93% based on proton shifts at 7.25 – 7.00 and 5.35.

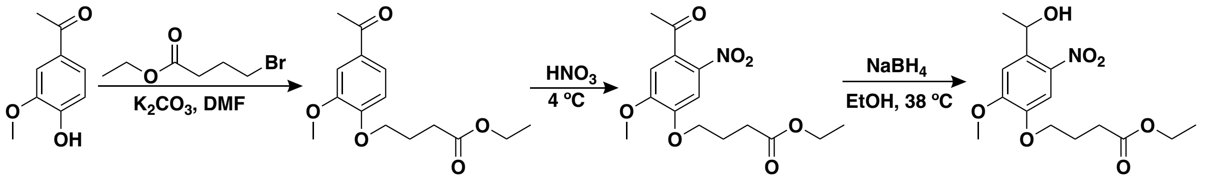

I II III IV

Figure S5. Scheme for the synthesis of **Compound IV**. Briefly, acetovanillone (**I**, 30 g, 180.5 mmol) was dissolved in dimethylformamide (DMF) (150 mL) followed by the addition of ethyl-4-bromobutyrate (31 mL, 217 mmol) and potassium carbonate (37.4 g, 271 mmol). The reaction was mixed overnight at room temperature under argon. The reaction mixture was precipitated in cold DI water (2000 mL), stirred for 2 h at room temperature, and allowed to precipitate at 4 ºC overnight. The precipitate was filtered and dried under vacuum for 48 h at 40 ºC to yield **Compound II**. Over one hour, half of **II** was (25 g, 89 mmol) was slowly added to cold nitric acid (70 mL, 4 ºC) on ice while monitoring to ensure the reaction temperature did not exceed 30 ºC. The reaction was allowed to proceed for an additional 30 min at 4 ºC followed by precipitation at 4 ºC overnight. Filtration and recrystallization in ethanol and drying in a vacuum oven were preformed to yield **Compound III**. To **III** (12.5 g, 55 mmol) in ethanol (150 mL) was added sodium borohydride (0.9 g, 24 mmol). The reaction proceeded overnight at 30 ºC followed by precipitation in cold DI water for 2h and precipitation overnight at 4 ºC. The precipitate was filtered to yield **IV**.

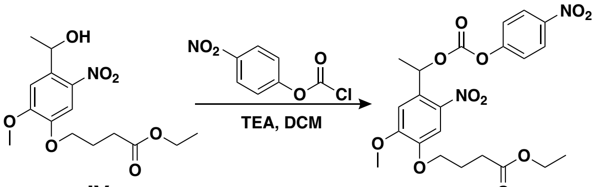

IV V

Figure S6. Scheme for the synthesis of **Compound V**. To anhydrous dichloromethane (DCM; 100 mL) at 0 ºC was added IV (3 g, 9.17 mmol) followed by pyridine (1.48 mL, 18.34 mmol), 4-dimethylaminopyridine (DMAP; 112 mg, 0.917 mmol), then 4-nitrophenyl chloroformate (1.87 g, 9.26 mmol). The reaction mixture was brought to room temperature and stirred overnight. Solvents were then removed under reduced pressure to yield a yellow crystalline slurry that was purified using flash chromatography (0% to 30% ethyl acetate: hexanes) to give **V**.

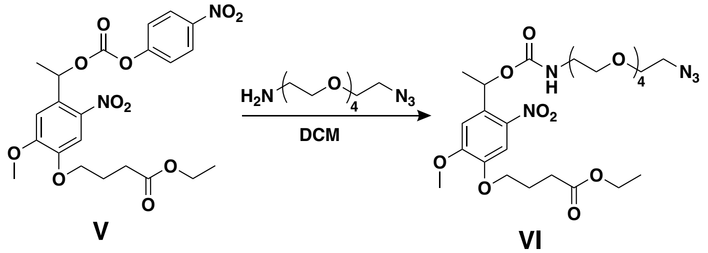

V VI

Figure S7. Scheme for the synthesis of **Compound VI**. Compound **V** (4 g, 8.13 mmol) was dissolved in DCM (80 mL) at 0 ºC followed by the addition of pyridine (1.43 mL, 16.3 mmol), DMAP (100mg, 0.813 mmol), and azido-tetraethyleneglycol-amine (2.15 g, 8.13 mmol) was added. The reaction mixture was left to stirred overnight. The solution was concentrated and purified via flash chromatography (0% to 75% ethyl acetate: hexanes) to produce **VI**.

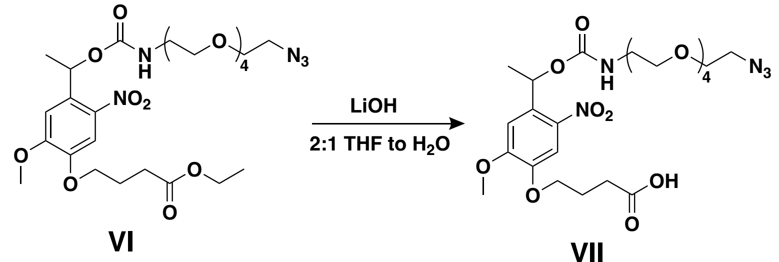

VI **VII**

Figure S8. Scheme for the synthesis of **Compound VII**. Compound **VI** (3.8 g, 6.2 mmol) was mixed with a 2:1 mixture of THF and DI water (75 mL) followed by the addition of a 1 M solution of lithium hydroxide (LiOH; 180 mg, 7.5 mmol). The solution was left to react overnight. To the reaction mixture 1 M HCl (100 mL) was added followed by DCM (300 mL). The organic phase was extracted and the aqueous phase was washed two additional times with DCM (100 mL). The organic phases were combined, dried over sodium sulfate (Na_2SO_4), concentrated, to yield **VII (Nitrobenzyl-azide)** without further purification.

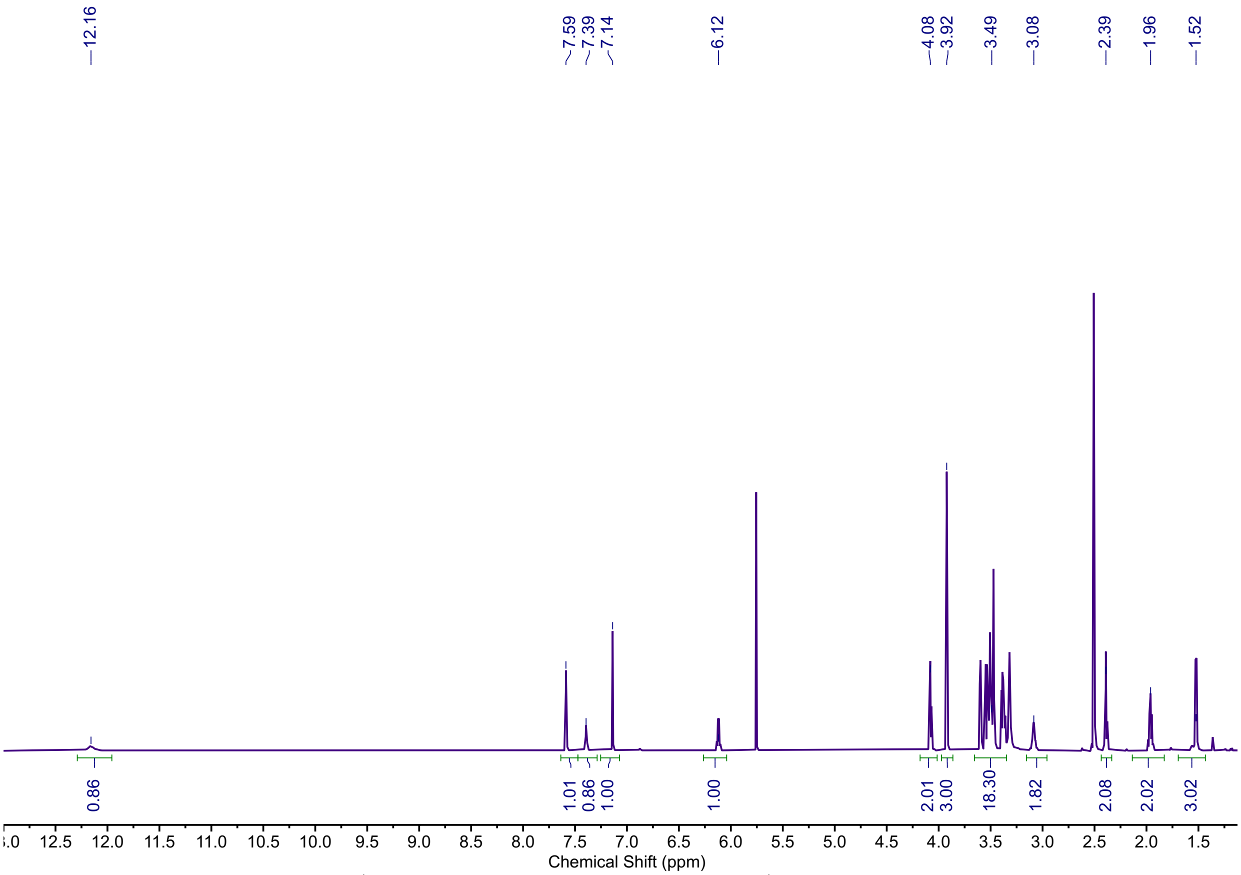

Figure S9. Representative ^{1}H NMR spectrum for NB-azide^{1}H NMR (600 MHz, DMSO) δ 12.16 (s, 1H), 7.59 (s, 1H), 7.39 (s, 1H), 7.14 (s, 1H), 6.12 (s, 1H), 4.08 (s, 2H), 3.92 (s, 3H), 3.49 (s, 18H), 3.08 (s, 2H), 2.39 (s, 2H), 1.96 (s, 2H), 1.52 (s, 3H).

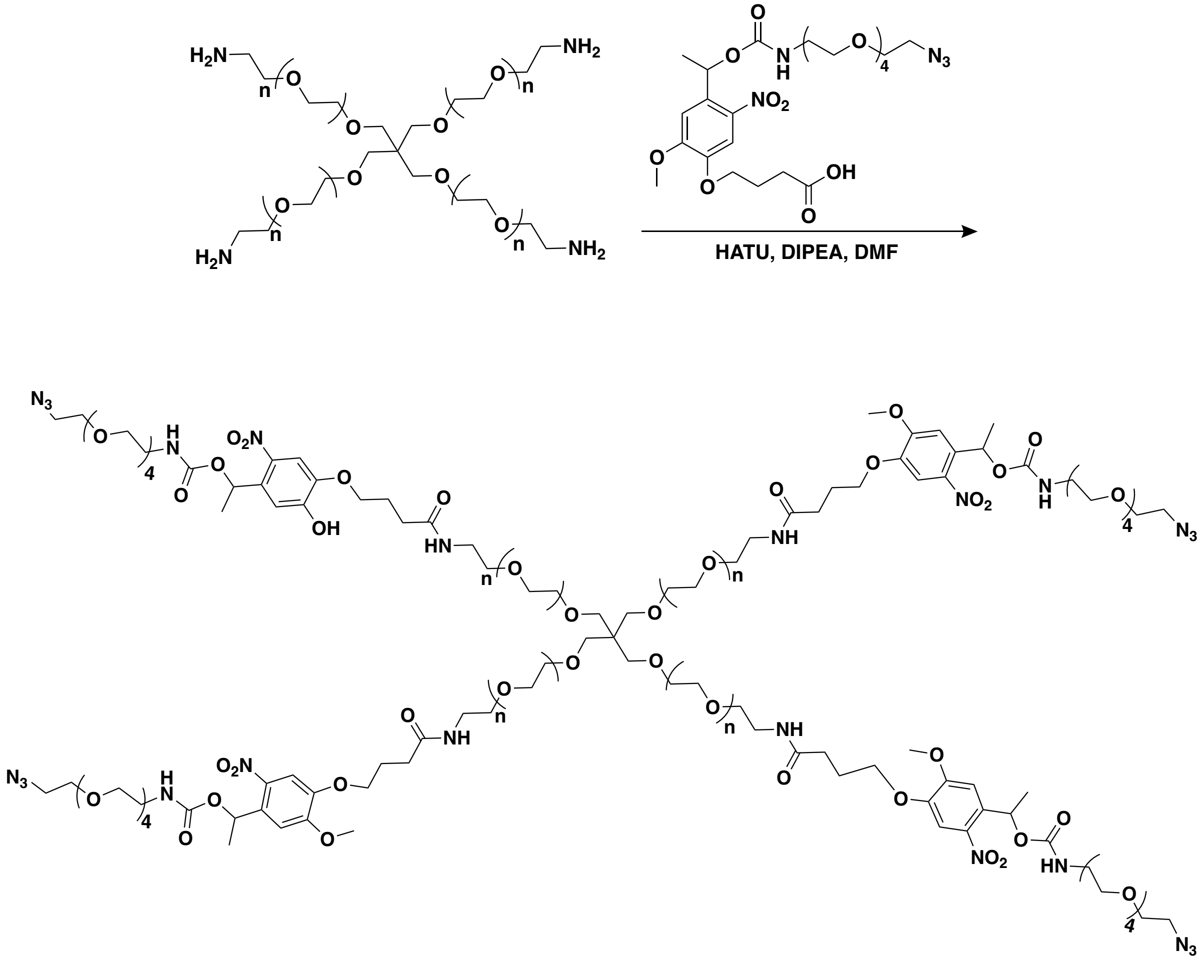

Figure S10. Scheme for the synthesis of PEG-4-NB-azide.

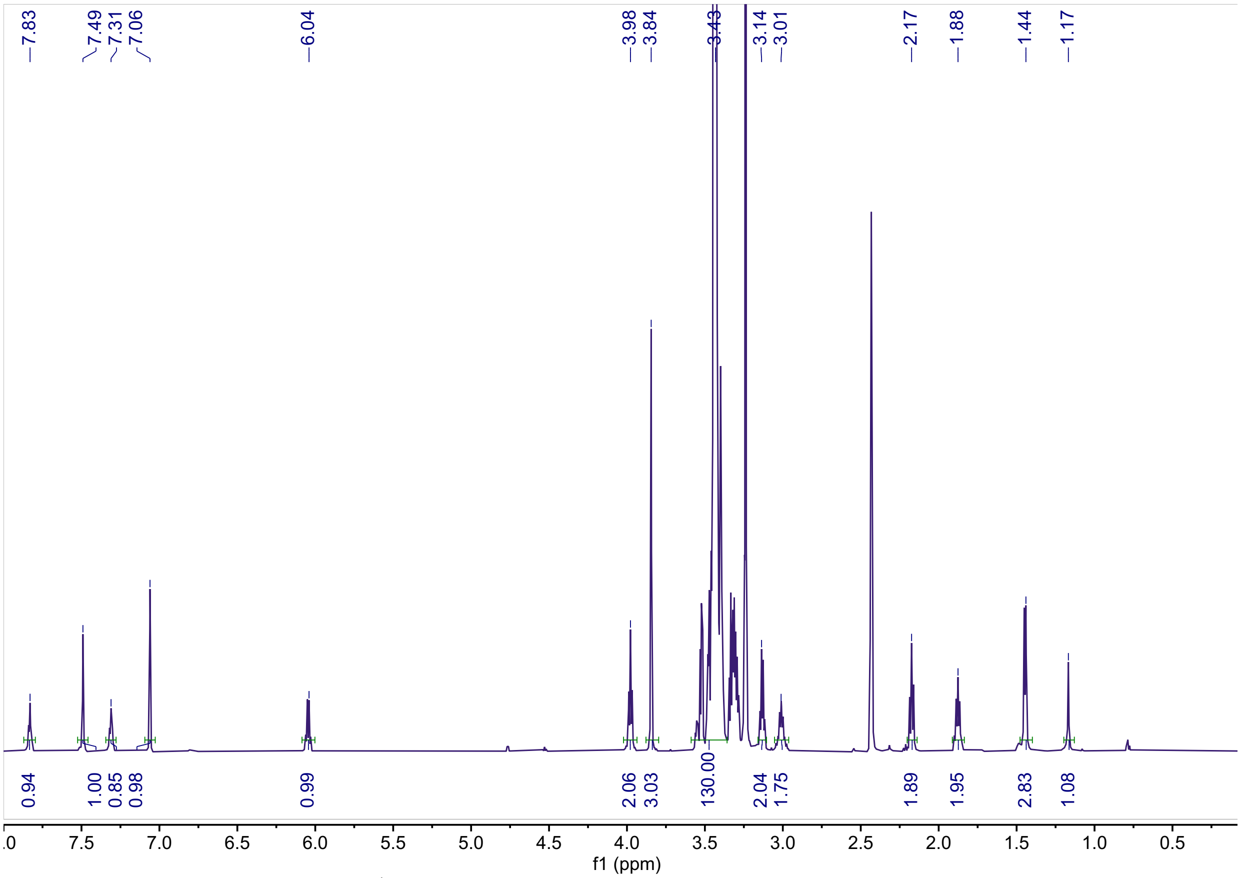

Figure S11. Representative ^{1}H NMR spectrum for PEG-4-NB-azide (600 MHz, DMSO-d_6, 128 scan) δ 7.83 (s, 1H), 7.49 (s, 1H), 7.31 (s, 1H), 7.06 (s, 1H), 6.04 (s, 1H), 3.98 (s, 2H), 3.84 (s, 3H), 3.50 (s, 130H), 3.14 (s, 2H), 3.01 (s, 2H), 2.17 (s, 2H), 1.88 (s, 2H), 1.44 (s, 3H), 1.17 (s, 1H). Functionality = 99% based on proton shifts at 7.83, 7.49, and 7.06.

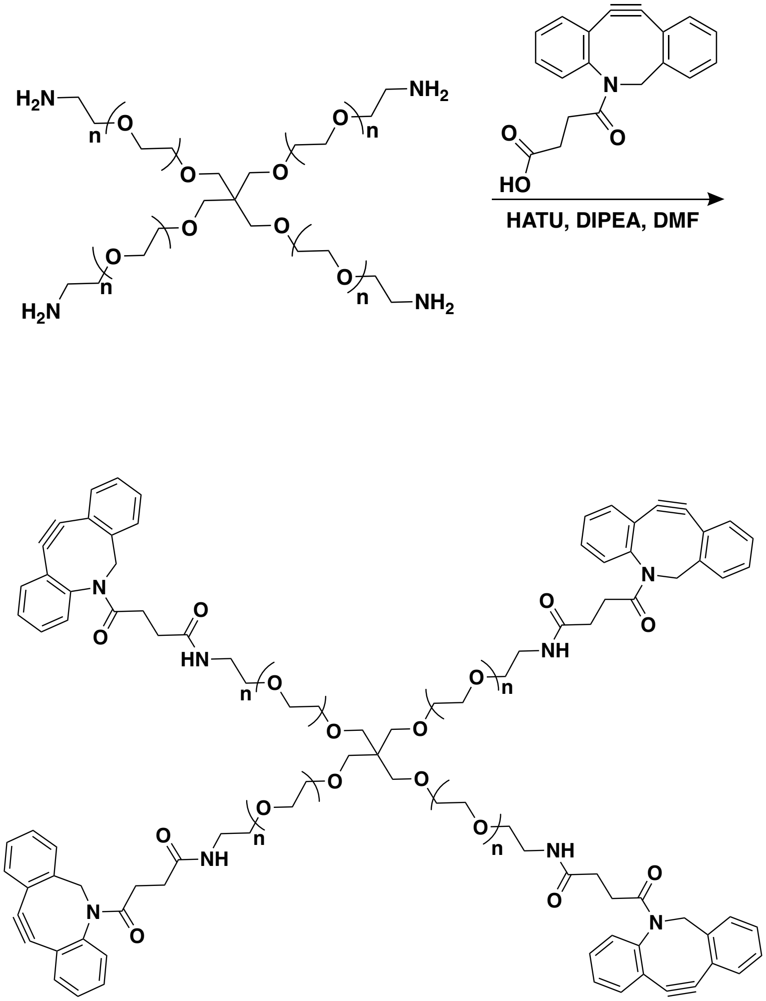

Figure S12. Scheme for the synthesis of PEG-4-DBCO.

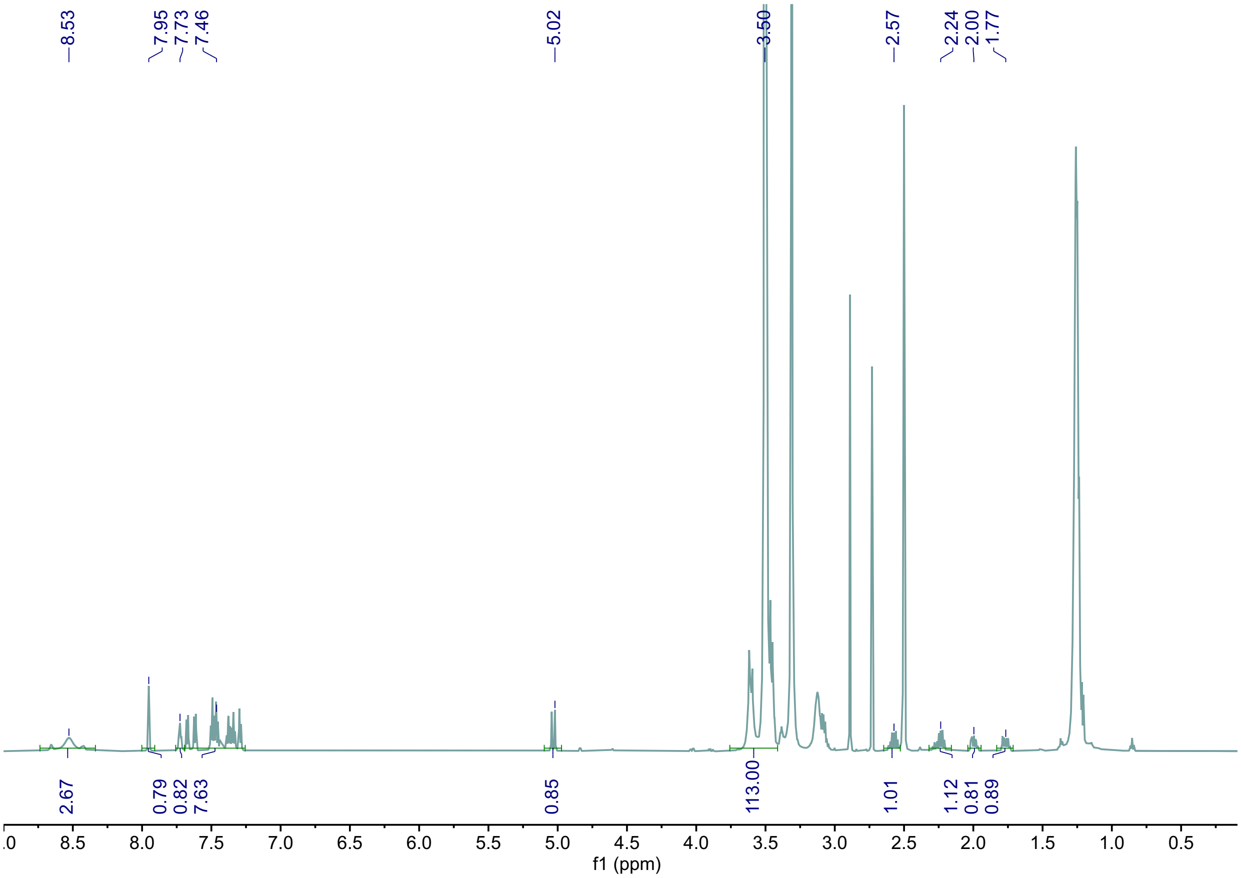

Figure S13. Representative ^{1}H NMR spectrum for PEG-4-DBCO (600 MHz, DMSO-d_6, 128 scan) δ 8.75 – 8.35 (m, 3H), 7.96 (s, 1H), 7.73 (t, *J* = 5.7 Hz, 1H), 7.70 – 7.26 (m, 8H), 5.04 (d, *J* = 14.0 Hz, 1H), 3.77 – 3.42 (m, 113H), 2.58 (dt, *J* = 16.0, 7.7 Hz, 1H), 2.24 (dt, *J* = 15.4, 7.7 Hz, 1H), 2.00 (m, 1H), 1.79 (dd, *J* = 8.4, 5.9 Hz, 1H). Functionality = 89% based on proton shifts at 7.70 – 7.26 and 5.04.

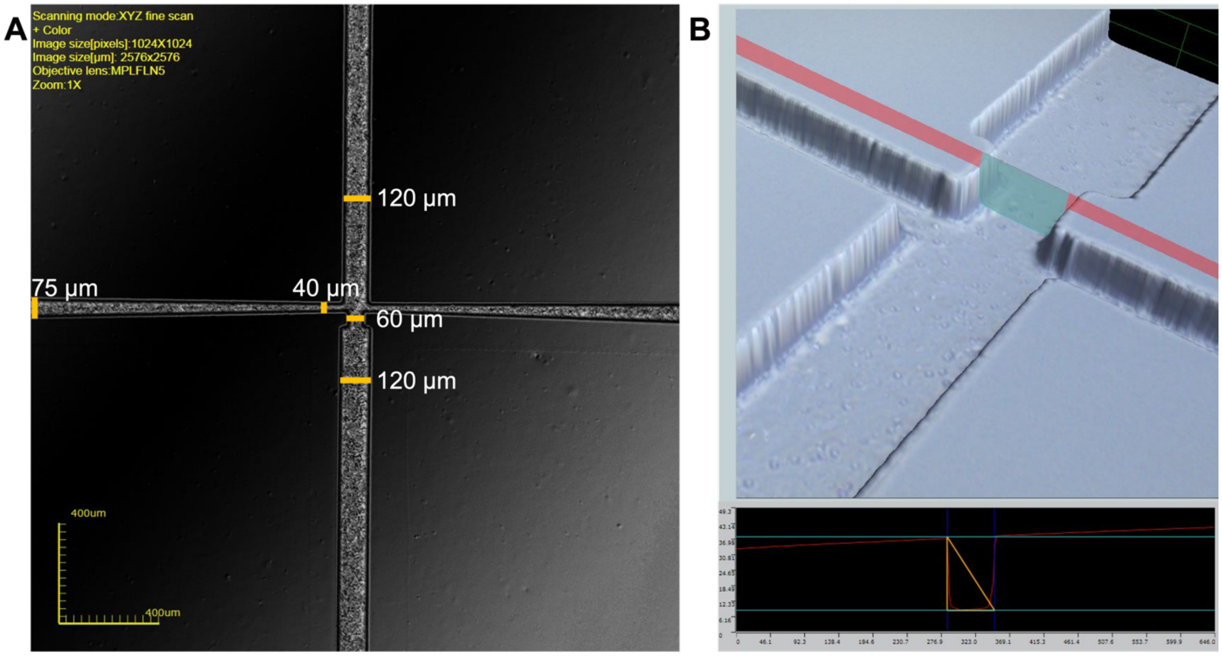

Figure S14. *MFF device design.* Scanning optical profilometry (LEXT OLS4000, Olympus) of the polydimethylsiloxane (PDMA) microfluidic replica used to produce microgels in this study (A) and a 3D enlargement of the flow focusing region with a 2D depth line-out to indicate cross sectional profile at the junction (B). Droplets were produced by merging aqueous hydrogel forming solutions from the center stream with fluorocarbon oil from both side streams passed through a junction with cross sectional dimensions of 30 µm x 60 µm (depth x width). All feed channels were tapered in order to maximize local velocity and therefore shear at the junction.

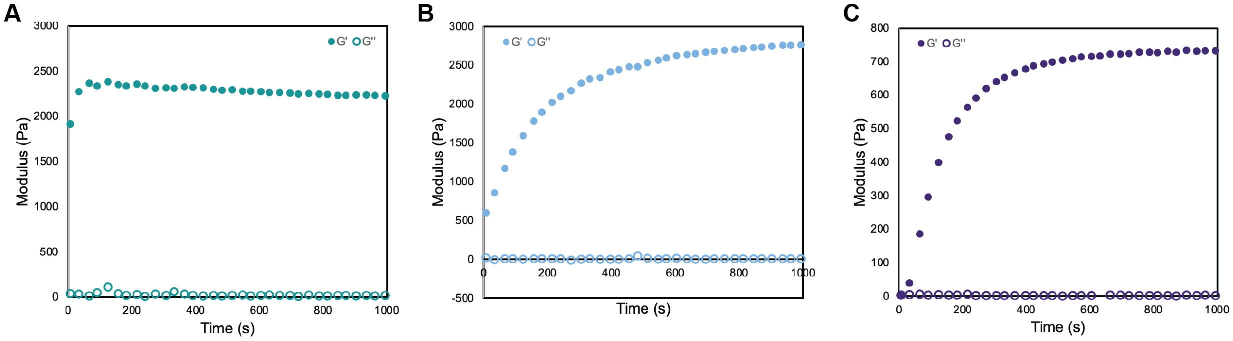

Figure S15. *Representative rheology time sweep plots.* Time sweep rheology plots for 90% redox-responsive (A), 50% redox-responsive (B), and light-responsive (C) microgel formulations investigated for microgel formation.

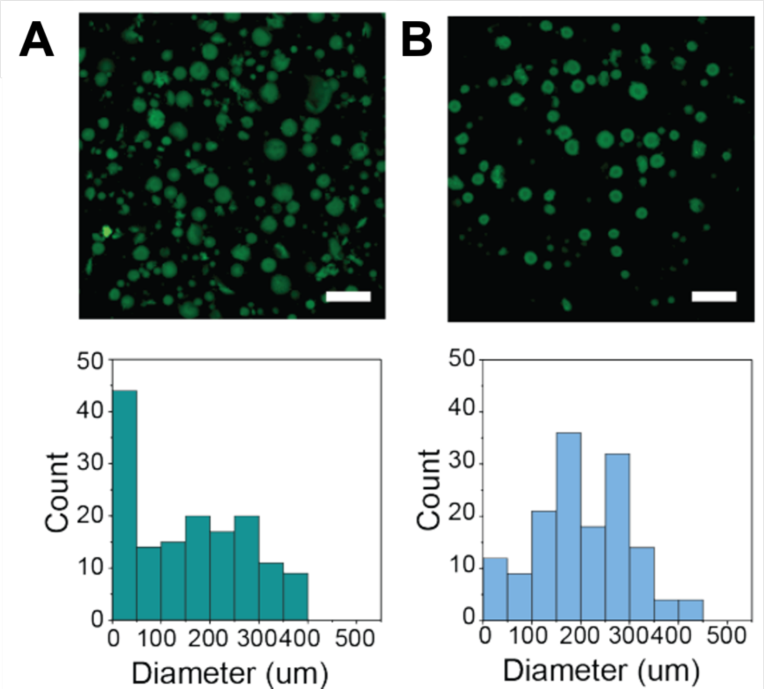

Figure S16. *Microgels formed using inverse suspension polymerization.* Microgels were formed using inverse suspension polymerization with PEG-4-arylSH:alkylSH and PEG-4-MI precursor components loaded with BSA-AF488. Microgels were formed with (A) 33% and (B) 66% PEG-4-arylSH. Microgels were observed to be highly disperse and larger in size than those formed using microfluidic flow focusing. Scale bars = 500 µm. Representative images shown on top with histograms showing the distribution of diameters on the bottom.

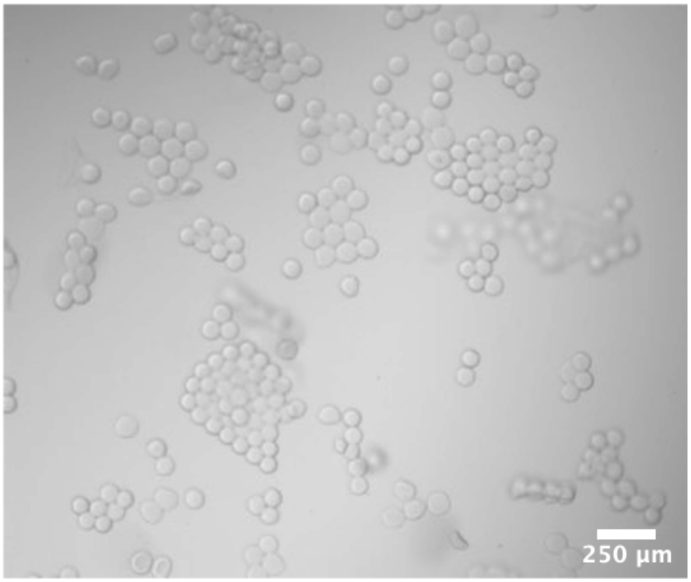

Figure S17. *Microgels injected through 30G needle.* Microgels with a diameter of approximately 75 μm were injected through a 30G blunt tip syringe needle without observable damage to the microgels. Microgels were imaged after being passed through the syringe needle using an inverted microscope with black and white camera (Zeiss). Scale bars 250 μm.
